# ECM remodeling-associated immune signatures and hub proteins: predictive markers and therapeutic targets for metastatic gastric cancer

**DOI:** 10.3389/fimmu.2026.1765095

**Published:** 2026-02-06

**Authors:** Xinyue Hu, Kai Zhao, Yaoqiang Shi, Zhongjian Liu, Yi Sun

**Affiliations:** 1 Faculty of Life Science and Technology, Kunming University of Science and Technology, Kunming, China; 2 The Affiliated Hospital of Kunming University of Science and Technology, Kunming, China; 3 Institute of Basic and Clinical Medicine, First People’s Hospital of Yunnan Province, Affiliated Hospital of Kunming University of Science and Technology, Kunming, Yunnan, China; 4 Neurosurgery Department, the Second Affiliated Hospital of Kunming Medical University, Kunming, China

**Keywords:** extracellular matrix (ECM), gastric cancer, metastasis, single-cell RNA sequencing, tumor

## Abstract

**Background:**

Gastric cancer (GC) remains one of the most prevalent malignancies worldwide. A growing number of studies have identified the extracellular matrix (ECM) as a key regulator in gastric cancer development and metastasis. Gastric cancer cells interact with the ECM, modifying its composition and structure, thereby promoting tumor invasiveness and metastatic ability. However, the specific molecular mechanisms underlying these processes remain poorly understood.

**Methods:**

In this study, we identified metastasis-related hub proteins in the extracellular matrix by proteomics, explored the differentiation trajectories of tumor cells at the single-cell levelidentified metastasis-related trajectories and metastasis-associated tumor cell subtypes, and experimentally verified the roles and mechanisms by which metastasis-associated ECM proteins regulate the migration and invasion of gastric cancer cells.

**Results:**

Our proteomic analysis revealed 282 prognosis-related differential proteins between gastric cancer metastasis-free and metastasis-associated groups, including 77 extracellular matrix proteins. We constructed a protein-protein interaction (PPI) network and identified four core hub proteins using network centrality measures. Single-cell RNA sequencing provided a detailed landscape of gastric cancer cells, enabling exploration of the expression patterns of these hub proteins across different cell subtypes. Trajectory analysis uncovered distinct pathways associated with tumor metastasis and highlighted the regulatory roles of hub proteins in this process. We identified two key tumor cell subtypes critical for metastasis, and through cell communication analysis, identified significantly enriched pathways linked to metastatic progression. *In vitro* experiments further validated the functional role of Ribosomal Protein S29(RPS29) in the metastatic mechanisms of gastric cancer cells.

**Conclusion:**

This study not only reveals the complexity of ECM remodeling in the microenvironment of gastric cancer but also provides new perspectives and potential targets for future gastric cancer treatment and brings opportunities for the development of new therapeutics, which are expected to overcome the challenge of cancer metastasis.

## 1 Introduction

Gastric cancer ranks as the fifth most common cancer in the world and is the third leading cause of cancer-related deaths worldwide (1). Gastric cancer patients frequently experience tumor metastasis, especially to the liver and peritoneum, which significantly reduces survival rates (2). Consequently, gaining a deeper understanding of the mechanisms driving gastric cancer metastasis is crucial.

The extracellular matrix (ECM) is a non-cellular component between cells, mainly composed of collagen, elastin, glycosaminoglycans, glycoproteins, and other components (3). Studies have shown that as cancer progresses, tumor cells undergo significant morphological and functional changes, largely driven by their dynamic interplay with the surrounding microenvironment (4). Specifically, alterations in ECM composition and stiffness can directly modulate intracellular signaling through integrin-mediated pathways, activating cascades such as FAK, Src, and Rho GTPases to promote cytoskeletal reorganization, epithelial-mesenchymal transition (EMT), and directed cell migration, all of which are hallmarks of invasive cancer (5–7).

The ECM plays an important role in the regulation of homeostatic processes, including cell proliferation, migration, and differentiation (8). In the context of cancer, alterations in the composition and structure of the ECM significantly influence tumor progression, metastasis, and patient prognosis (9). The ECM promotes tumor cell invasion and proliferation through multiple mechanisms (10). One key aspect is the ability of tumor cells to secrete specific enzymes that degrade ECM components. This degradation enhances the cells’ migratory capacity and invasiveness, enabling them to breach surrounding tissue barriers and enter the vascular or lymphatic systems, thereby promoting metastasis (11).

RPS29, a structural component of the small ribosomal subunit, plays a crucial role in cellular gene expression, RNA processing and transport, cell cycle regulation, and cell proliferation. While research on RPS29 in tumors is limited, studies have shown that its downregulation significantly inhibits ribosomal gene expression, protein synthesis initiation, and the growth of triple-negative breast cancer cells (12). Other RPS family members have been more extensively studied in cancer. For instance, RPS27 is elevated in prostate cancer and associated with poor prognosis, suggesting its potential as a diagnostic and therapeutic target (13). RPS6 overexpression is linked to tumor malignancy and patient prognosis in various cancers, and its absence inhibits cholangiocarcinoma cell proliferation and tumor growth by disrupting the spliceosome complex (14). Moreover, RPS15 promotes esophageal squamous cell carcinoma via m6A modification, offering a potential therapeutic strategy (15), and RPS3 is associated with clear cell renal cell carcinoma proliferation and metastasis (16), as well as colon cancer development through NF-κB signaling pathway regulation (17), suggesting its potential as a diagnostic and therapeutic target in these cancers. Importantly, RPS29’s role in gastric cancer has not been previously investigated.

While bulk proteomic studies have identified broad changes in ECM composition in GC, a critical knowledge gap remains in understanding how these changes functionally translate to the behavior of specific tumor cell subpopulations. Bulk analyses average signals across a heterogeneous cell population, obscuring the contributions of rare but potentially crucial cell phenotypes that initiate the metastatic cascade (18, 19). It remains poorly understood which specific tumor cell subtypes are primarily responsible for responding to or remodeling the pro-metastatic ECM and what transcriptional programs govern their progression.

The primary limitation of conventional bulk-omics approaches is the averaging of molecular signals across millions of diverse cells, which masks the contributions of distinct cellular subpopulations. This is particularly problematic in cancer, where a small population of aggressive, stem-like cells may be solely responsible for driving metastasis (20). Single-cell technologies, such as scRNA-seq, overcome this fundamental limitation by providing a high-resolution transcriptional snapshot of every individual cell within the tumor ecosystem. This allows us to computationally deconstruct tumor heterogeneity, identify rare but clinically relevant cell subtypes, uncover novel marker genes, and reconstruct cellular differentiation and progression pathways (21).

Our study integrated proteomics, single-cell transcriptomics, and *in vitro* experiments to systematically identify key hub proteins within the extracellular matrix (ECM) associated with gastric cancer metastasis and prognosis. Notably, we experimentally validated for the first time the promoting role of ribosomal protein RPS29 in gastric cancer metastasis and its regulation of epithelial-mesenchymal transition (EMT). This provides a novel molecular perspective for understanding gastric cancer metastasis and suggests that RPS29 may represent a potential intervention target.

## 2 Materials and methods

### 2.1 Proteomics of tumors to identify key extracellular matrix proteins promoting metastasis

#### 2.1.1 Data acquisition

1. We obtained proteomics data from the **Supplementary Materials** (**Supplementary Data 1**, **Data 2**) of a previous study (PMCID: PMC9522856 (22)). This dataset comprised a total of 110 samples (**Table 1**).
2. Single-cell datasets GSE163558 (23)and GSE167297 (24)were downloaded from the Gene Expression Omnibus (GEO) database (https://www.ncbi.nlm.nih.gov/geo/query/acc.cgi). From GSE163558, three primary tumor samples were selected: two with metastasis and one without. From GSE167297, samples representing deep invasive layers were selected, and based on pathological metastasis (pM) clinical data, two samples with metastasis and three without were retained. The integrated samples were subsequently renumbered for this study (**Table 2**).
3. The bulk RNA-sequencing data for stomach adenocarcinoma (TCGA-STAD) were downloaded from the UCSC Xena database (25)(http://xena.ucsc.edu/) (**Table 3**).

**Table 1:** Gastric cancer proteomics sample information.

Patients Information	PMC9522856
age	
>50	17
<=50	93
grade	
I	4
II	22
III	84
pM	
M0	90
M1	20
pN	
N0	30
N1-3	70
NX	10
pT	
T0	8
T1	8
T2	14
T3	27
T4	42
TX	11
OS	
Alive	82
Dead	28

**Table 2:** Single-cell data sample information.

DataSet	SampleID	Condition	ID in this study
GSE163558	PT1	liver metastasis	M1
	PT2	no metastasis	T1
	PT3	ovary metastasis	M2
GSE167297	P1 (Deep)	no metastasis	T2
	P2 (Deep)	no metastasis	T3
	P3 (Deep)	peritoneal metastasis	M3
	P4 (Deep)	metastasis	M4
	P5 (Deep)	no metastasis	T4

**Table 3:** Prognostic and Clinical Information of TCGA-STAD Data.

Patients Information	TCGA-STAD
pM	
M0	327
M1	25
OS	
Alive	214
Dead	138
DFI	
Alive	171
Dead	36
NA	145
DSS	
Alive	247
Dead	86
NA	19
PFI	
Alive	233
Dead	119

#### 2.1.2 Differential extracellular matrix proteins and enrichment analysis

Extracellular matrix proteins were primarily sourced from the Molecular Signatures Database (MSigDB) database (22) (https://www.gsea-msigdb.org/gsea/msigdb/index.jsp). Six relevant Gene Ontology (GO) terms related to the extracellular matrix were identified through a search of the database. The genes associated with these terms were then extracted for subsequent analysis (**Table 4**).

**Table 4:** Extracellular matrix gene acquisition entries.

Terms	Number of Genes
GOCC_ANCHORING_JUNCTION	901
GOCC_BASEMENT_MEMBRANE	90
GOCC_CELL_SUBSTRATE_JUNCTION	434
GOCC_COLLAGEN_CONTAINING_EXTRACELLULAR_MATRIX	430
GOCC_INTERSTITIAL_MATRIX	11
GOCC_MICROFIBRIL	13

Following the methodology outlined in a previous study (PMC9522856), we began by excluding histological samples lacking clear pathological metastasis grouping and associated prognostic information. To mitigate potential calculation errors arising from the high prevalence of zero expression values, we initially filtered the protein dataset, retaining only those proteins expressed in at least one-third of the samples. Subsequently, we employed the Wilcoxon test to identify differential protein expression between metastasis-positive and metastasis-negative groups. Volcano plots were generated to visualize these differences. Differentially expressed proteins (DEPs) were identified using thresholds of *p* < 0.05 and an absolute log2 fold change (|log2FC|) greater than 0.5. GO and Kyoto Encyclopedia of Genes and Genomes (KEGG) pathway enrichment analyses were then performed using the clusterProfiler (26) R package (v4.6.2) on the genes corresponding to both the up- and down-regulated DEPs. The results of these enrichment analyses are presented as bar charts.

#### 2.1.3 Hallmark pathway enrichment analysis

Hallmark enrichment scores were calculated for samples grouped by pathological metastasis status using the ssGSEA() function from the GSVA (27) R package (v1.34.0). Heatmaps were then generated for visualization. Subsequently, pathways exhibiting statistically significant differences between groups (Wilcoxon test, *p* < 0.05) were identified. Differential expression was assessed using the Wilcoxon rank-sum test. P-values were adjusted for multiple testing using the Benjamini-Hochberg method (False Discovery Rate, FDR). A significance threshold of adjusted p-value (FDR) < 0.05 was used. For each of these pathways, the optimal cutpoint for patient stratification was determined using the surv_cutpoint of the survival (v3.2-7) function from the survival R package (v3.2-7). This function divided patients into high and low subgroups based on pathway enrichment scores. Finally, Kaplan-Meier (KM) curves were generated and plotted for these subgroups using the survminer R package (v0.4.8) for presentation.

### 2.2 Identification of ECM hub proteins driving tumor metastasis

Initially, we identified differentially expressed proteins between groups with different pathological metastasis statuses. Simultaneously, we conducted a prognostic analysis across all proteins to identify those associated with prognosis. The intersection of these two protein sets yielded the prognosis-related differentially expressed proteins, from which we selected the top 50 for further analysis. Next, we determined the intersection between prognosis-related proteins and extracellular matrix-related proteins, thereby identifying the prognosis-related extracellular matrix proteins. Using the top 50 prognosis-associated differentially expressed proteins and the prognosis-associated extracellular matrix proteins, we constructed a protein interaction network. This network was visualized using Cytoscape (28) (v3.9.1), and its parameters were subsequently analyzed with the cytoHubba plugin. From the network, we extracted the top ten genes based on Degree, Closeness, and Betweenness centrality measures. The intersection of these genes with the top 50 prognosis-related differentially expressed proteins revealed the core hub proteins. Finally, we generated box plots and prognostic curves to illustrate our findings.

### 2.3 Single-cell transcriptome analysis hub protein landscape

#### 2.3.1 Single-cell transcriptome landscape

To analyze the data, we employed the R package Seurat (v4.1.1). Our analysis began with quality control, using data from two datasets. Specifically, we filtered cells based on feature data (nFeature_RNA, nCount_RNA, and percent.mt), removing those with fewer than 200 expressed genes (nFeature_RNA) or a mitochondrial RNA proportion (percent.mt) exceeding 20%. We visualized these features using scatter plots and violin plots. Following quality control, we normalized the expression data for each sample’s cells using the NormalizeData() function. Subsequently, we identified highly variable genes (the 2000 genes exhibiting the greatest expression differences between cells by default) within each sample using the FindVariableFeatures() function. Recognizing the presence of batch effects between samples, we performed integration. This involved using FindIntegrationAnchors() to identify anchors and IntegrateNormalizedData() to integrate the normalized data, specifying dims = 50. We then scaled the expression values using the ScaleData() function and performed linear dimensionality reduction with RunPCA(), based on the highly variable genes and the first 20 principal components (PCs). Uniform Manifold Approximation and Projection (UMAP) dimensionality reduction was performed using RunUMAP ()(top 20 PCs), and sample-based UMAP plots were generated for visualization. A shared nearest neighbor (SNN) graph was constructed using FindNeighbors(), based on the overlap of each cell’s proximity to other cells. Cell subtype identification was performed using FindClusters() with a resolution of 0.08 and the top 20 PCs. Differential gene expression analysis between identified subtypes was conducted using FindAllMarkers() with thresholds of logfc.threshold = 0.25 and an adjusted *p*-value (*p*_val_adj) less than 0.05. Using these differentially expressed genes as subtype-specific markers, we performed cell type identification. This was achieved by comparing our markers with those reported in the literature and the CellMarker 2.0 database (29) (http://bio-bigdata.hrbmu.edu.cn/CellMarker/).

Finally, UMAP visualization was performed after cell type identification.

The processed data were saved as.rds files for subsequent analysis. Subsequently, bubble plots and UMAP plots were generated to visualize the expression levels of the characterized genes across different cell types, using the DotPlot() and FeaturePlot() functions. Meanwhile, we calculated the difference in the proportion of relevant cell types between the metastatic and non-metastatic subgroups, presenting these findings in box plots.

#### 2.3.2 Subtype analysis

To explore the relationship between hub proteins and individual cell types, we first calculated core protein scores for each hub protein’s corresponding genes using the AddModuleScore() function within the Seurat package. We then divided cells into high- and low-scoring subgroups based on the median score and visualized the score and subgroup distributions using UMAP plots. Finally, we used box plots to demonstrate statistically significant score differences between metastatic and non-metastatic subgroups. Due to the limited number of hub proteins, Non-negative Matrix Factorization (NMF) could not be used for cell subtype identification. However, given the substantial number and heterogeneity of T/NK cells, myeloid cells, epithelial cells, and fibroblasts, we proceeded with subtype identification for these four cell types using established cell subtype markers. We then generated UMAP plots to visualize hub protein expression differences across the identified cell subtypes. These plots illustrate the expression of hub proteins within each subtype. Moreover, box plots were generated to compare hub protein scores across all cell types and subtypes, stratified by metastatic status.

#### 2.3.3 Functional enrichment analysis

Using the KEGG gene set and the UCell algorithm within the R package irGSEA (v2.1.5) (30), we calculated metabolic signature-related scores for each cell and subtype. To visualize these results, we generated box-and-line plots displaying the top 20 pathways exhibiting the greatest differences in pivotal protein scores among subgroups. Besides, we generated bubble plots to illustrate the top 20 pathways with the largest differences in pivotal protein scores across different cell types.

### 2.4 Characterization of tumor cell functional features and identification of stem cell subclusters

#### 2.4.1 Expression of HLA genes and co-stimulatory molecules

Two independent studies provided the gene sets used in this analysis: T cell co-stimulation-related genes (PMID: 37068318) (31) and HLA genes (PMID: 35533264) (32). Based on the expression of the tumor marker EPCAM, we inferred that the Proliferative cells1 and Tumor_cells within the Epithelial cell population were malignant. Consequently, we generated heatmaps to visualize the expression of the identified genes in these two types of cells. Heatmaps displaying the expression of these genes across all cell types are provided in **Supplementary Figure 9**. Furthermore, we analyzed the distribution of all cells within subgroups defined by the presence or absence of metastasis and also visualized the distribution of all cells across the different samples.

#### 2.4.2 Relationship between hub proteins and inferCNV subgroups

Malignant cells are often characterized by large-scale chromosomal abnormalities. inferCNV is a tool designed to detect such copy number variants (CNVs), including amplifications or deletions affecting entire chromosomes or large chromosomal segments, within tumor single-cell RNA sequencing (scRNA-Seq) data. The method compares gene expression intensity in each tumor cell to a baseline of average gene expression from normal reference cells across the genome. Given that epidermal cells, endothelial cells, and fibroblasts are common origins of malignant cells in cancer, we performed an initial inferCNV analysis [R package InferCNV (v1.14.2)] using these cell types as a reference without specifically identifying CNV isoforms. This initial analysis aimed to assess the expression intensity of Proliferative cells and tumor cells, both considered malignant in this context. As these cell types were predominantly found in the M1 and P1 samples, subsequent, separate inferCNV analyses were conducted on each of these samples. These analyses employed the Hidden Markov Model (HMM) algorithm to predict CNV changes and identify CNV subtypes within each sample. Subsequently, the uphyloplot2 (33) package in Python was used to construct a clonal tree based on the identified subtypes. Branch lengths on this tree represent the proportion of each subtype. To annotate significantly enriched CNV variations within each sample, we integrated gene expression heatmaps of different chromosomal regions with branch frequency statistics. The statistical significance of enrichment within each branch was assessed using a chi-square test. Finally, UMAP plots were generated to visualize the distribution of each branch within the samples and were compared with the expression of hub proteins.

### 2.5 Exploration of the tumor hub protein-related regulatory modes and their mechanisms driving metastasis

#### 2.5.1 Differentiation and development of tumor subtypes

To further investigate the distinction between proliferating and tumor cells, a time-series analysis was conducted using the R package Monocle (v2.28.0), as the single-cell dataset lacked raw data, precluding RNA rate analysis. CellDataSet (CDS) objects were initially constructed using the newCellDataSet() function. Subsequently, size factors and dispersions were calculated with estimateSizeFactors() and estimateDispersions(), respectively. Highly variable genes from both cell subtypes served as feature genes for marker-based sorting via setOrderingFilter(). Dimensionality reduction was then performed using the DDRTree algorithm implemented in reduceDimension(). Leveraging the Progenitor Cell Biology Consortium (PCBC) database, the one-class logistic regression (OCLR) algorithm trained a stemness index, which was subsequently used to calculate a stemness score for each cell based on Spearman correlation. The state exhibiting the highest stemness score was designated as the trajectory’s starting point. The root of the pseudotime trajectory was defined as the cell population with the highest stemness score. This score was calculated using a gene signature derived from mouse embryonic stem cells (mESCs) from the PCBC database. Finally, cellular ordering and trajectory construction were completed using the function.

Following the acquisition of cell trajectories, these trajectories were initially visualized according to pseudotime, cell state, metastatic status, and cell subtype. These visualizations were then integrated with metastasis trajectories to identify cell states associated with tumor metastasis. Subsequently, the expression of key proteins within these trajectories was investigated, and time-series scatter plots and heatmaps were generated for illustrative purposes. Differential gene expression analysis was performed using the differentialGeneTest() function on the cellular mimetic time series, employing thresholds of at least 100 expressing cells and a q-value less than 0.05. The 500 most significantly differentially expressed genes were selected to construct a trajectory heatmap, enabling the identification of distinct gene expression subtypes. Finally, KEGG functional enrichment analysis was conducted, and the correlation between the expression of related genes and the key proteins was assessed, with a correlation heatmap generated for visualization.

Cell cycle stage prediction was performed for each cell using the CellCycleScoring() function from the Seurat package. Subsequently, bar graphs were generated to illustrate the percentage of cells in each cell cycle stage across various cell types and subgroups, specifically those defined by the presence or absence of metastasis.

#### 2.5.2 Bulk prognostic analysis

To investigate the prognostic value of metastasis-associated tumor cells, distinct cell phases within various cell subtypes were analyzed. Feature genes for each cell subtype and phase were identified using FindAllMarkers(), and the top 100 feature genes were selected. ssGSEA was employed to calculate the enrichment scores for each sample’s cell subtype and phase. Samples were then categorized into high- and low-scoring groups based on the median score. Kaplan-Meier survival curves were generated using overall survival (OS), disease-specific survival (DSS), disease-free interval (DFI), and progression-free interval (PFI) to determine the prognostic significance of the identified cell features.

### 2.6 Differential activation of tumor metastasis-related transcription factors

Constructing a gene regulatory network involved several key steps. First, potential target genes for each transcription factor were identified through co-expression analysis, using a gene expression matrix and algorithms like GENIE3/GRNBOOST to generate co-expression modules. Subsequently, DNA motif analysis was performed to pinpoint potentially direct targets, which requires defining cellular states and their respective regulators. Network activity was then analyzed by scoring regulators within each cell, calculating the area under the curve (AUC), and transforming this activity into a two-dimensional matrix. Finally, the Python package pysceinc was employed to identify metastatic intergroup-specific transcription factors and construct the gene regulatory network.

Following the arithmetic process, the cell’s transcription factor activity data were stored in the pyscenic_output.rds file, from which the area under the curve (AUC) value for each transcription factor was derived. These AUC values were then used to calculate the Connection Specificity Index (CSI) for each transcription factor. Based on the CSI, transcription factors were grouped into modules and visualized with a heatmap displaying CSI values across modules. Furthermore, UMAP and time series plots illustrated the mean CSI value for each module’s transcription factors. Subsequently, the Regulon Specificity Score (RSS) was calculated based on metastasis grouping, visualized with an RSS ranking diagram. UMAP plots then displayed the AUC of the top 10 regulons for both metastasis and non-metastasis groups. Finally, functional enrichment analysis of these top 10 regulons was performed using the clusterProfiler R package, with enriched pathways (*p*-value < 0.05 and q-value < 0.2) presented in a bubble plot.

### 2.7 Characterizing cellular communication differences between primary and metastatic cancer using single-cell data

The R package CellChat (34) (v 1.6.1) was further utilized for communication analysis of all cells. Single-cell data were first stratified into metastasis and non-metastasis groups, and CellChat objects were constructed for each group using the createCellChat() function with CellChatDB.human as the ligand-receptor database. Differentially expressed genes and ligand-receptor interactions were identified using the identifyOverExpressedGenes() and identifyOverExpressedInteractions() functions, respectively. Gene expression data were then projected onto a protein-protein interaction network using projectData(). Communication probabilities were calculated, and cell-cell communication networks were extrapolated with computeCommunProb(). A minimum cell threshold of 10 (min.cells = 10) was applied using filterCommunication(). Signaling pathway-level communication was inferred with computeCommunProbPathway(), and finally, the resulting cell-communication networks were integrated using aggregateNet().

After obtaining the communication information, enriched pathways between groups were compared using Fisher’s exact test to identify significant differences (*p* < 0.05). These differences were visualized using box plots. Subsequently, the top four significantly enriched pathways for each group were selected for circular plot representation. Moreover, network centrality indices were calculated for each cell population to determine their respective roles, and the corresponding importance was illustrated using a heatmap.

### 2.8 Identification of drugs that potentially inhibit metastasis

Enrichment scores for metastasis-related tumor cell subtypes were calculated using TCGA-STAD data, and samples were categorized into high and low-score groups based on the median enrichment score. Subsequently, samples were similarly divided into high and low-expression groups based on median hub protein expression. Drug response predictions were generated using the oncoPredict (35)(v0.2) R package, leveraging the CTRP2 and PRISM drug response databases. CTRP2 data were included with the oncoPredict package, while PRISM data were downloaded from the DepMap Portal (https://depmap.org/portal/). Gastric cancer cell data from both databases were processed, and drugs common to both were used for IC_50_ prediction. Wilcoxon tests were performed to compare IC_50_ values between high and low enrichment scores and expression groups. Drugs exhibiting consistent response trends across both databases and groupings, with statistically significant differences (*p* < 0.05), were visualized using box plots. These plots illustrated IC_50_ differences between high and low groups, as well as correlations with both enrichment score and hub protein expression. Finally, Spearman correlations between hub protein expression levels were visualized using scatter plots.

In addition, the Drug Gene Interaction Database (DGIdb) (36)was queried to identify gene pairs interacting with differentially expressed drugs. After selecting approved interactions, expression matrices for the interacting genes were extracted. Spearman correlations were calculated between these genes and drug IC_50_ values, enrichment scores, and hub protein expression levels. Bubble plots were then generated to visualize gene interactions within enrichment score subgroups, highlighting expression differences between hub protein expression subgroups.

### 2.9 Experimental validation of gastric cancer cells

#### 2.9.1 Cell lines and cell culture conditions

Human gastric cancer cell lines (AGS, HGC27, and MKN45) and the normal gastric mucosal epithelial cell line (GES-1) were obtained from Starfish Biologicals. AGS cells were cultured in Ham’s F-12 medium supplemented with 10% fetal bovine serum, while the other cell lines were cultured in 1640 medium supplemented with 10% fetal bovine serum. All cell lines were maintained at 37 °C in a 5% CO_2_ atmosphere. All cells are identified using STR cell identification and tested for Mycoplasma contamination.

#### 2.9.2 siRNA transfection

The siRNAs targeting RPS29 for HGC27 and AGS cell lines were synthesized by Shanghai Gemma Bio, with the following sequences: RPS29-siRNA1 (sense: GUCGUGUCUGUUUCAAACCGTT; antisense: CGGUUUUGAACACAGACACACGACTT) and RPS29-siRNA2 (sense: ACGCGAAGGAUAUCGGUUUUTT; antisense: AAACCGAUAUCCUUCGCGUTT). Transient transfection of HGC27 and AGS cells was performed using Gemma’s siRNA-mate plus transfection reagent, following the manufacturer’s recommended protocol for RPS29 knockdown.

#### 2.9.3 Wound healing assay

Scratch assays were performed by seeding cells in 12-well plates, adding 75 μL of cell suspension to each well, and incubating overnight at 37 °C until cells reached 95% confluence. Following scratch creation and removal of the inserts, cells were washed with phosphate-buffered saline to remove any detached cells. Serum-free medium was then added to inhibit further proliferation. The progression of wound closure was documented using an inverted microscope at 0, 12, and 24 hours.

#### 2.9.4 Transwell migration and invasion experiments

Transwell migration and invasion experiments were performed using 24-well chambers (Corning, 3422), with invasion assays employing a Matrigel coating (Corning, 356234). Cells were resuspended in a serum-free medium at a concentration of 3×10^5^ cells/mL to suppress proliferation. Subsequently, 200 µL of this cell suspension was introduced into the upper Transwell chamber, while 600 µL of medium supplemented with 15% FBS was added to the lower chamber. Following a 24-hour incubation period at 37 °C, cells remaining in the upper chamber were fixed with 4% paraformaldehyde and stained with 0.1% crystal violet. Non-migrated cells on the upper chamber’s surface were carefully removed with a cotton swab. Images were captured using an inverted microscope, and the number of migrated or invaded cells was determined using ImageJ software.

#### 2.9.5 Western blot

Cell lysis was performed using a RIPA lysis buffer (Beyotime, China, P0013B) supplemented with protease and phosphatase inhibitors, following the manufacturer’s instructions. Protein concentration was determined using the BCA protein assay kit (Beyotime, China, P0010). Equal amounts of proteins were then separated by SDS-PAGE (Tris-HCl) and transferred onto a PVDF membrane. Following a 2-hour blocking step with 5% skimmed milk at room temperature, the membranes were incubated overnight at 4 °C with the following primary antibodies: RPS29 (Proteintech, 17374-1-AP), β-actin (Servicebio, ZB15001-HRP), E-Cadherin (Abcam, ab314063), N-Cadherin (Abcam, ab76011), and Vimentin (Abcam, ab92547). Afterward, the membranes were incubated with an HRP-labeled secondary antibody for 2 hours at room temperature. Chemiluminescent signals were detected using enhanced chemiluminescence (ECL) and subsequently quantified using ImageJ software (NIH, Bethesda, USA).

#### 2.9.10 Statistical analysis

Statistical analysis was performed using GraphPad Prism 8.3 and R Studio software. All experiments were repeated at least three times, and results were expressed as mean ± standard deviation (SD). To determine statistical significance between groups, we employed the Kruskal-Wallis test, the Wilcoxon rank sum test, and the t-test, as appropriate. Survival differences were assessed using Kaplan-Meier analysis with the log-rank test. Pearson or Spearman correlation methods were used to evaluate the relationship between gene expression levels. A *p*-value <0.05 was considered statistically significant.

## 3 Results

### 3.1 Differential extracellular matrix proteins and enrichment analysis, identify ECM hub proteins driving gastric cancer metastasis.

**Figure 1:**
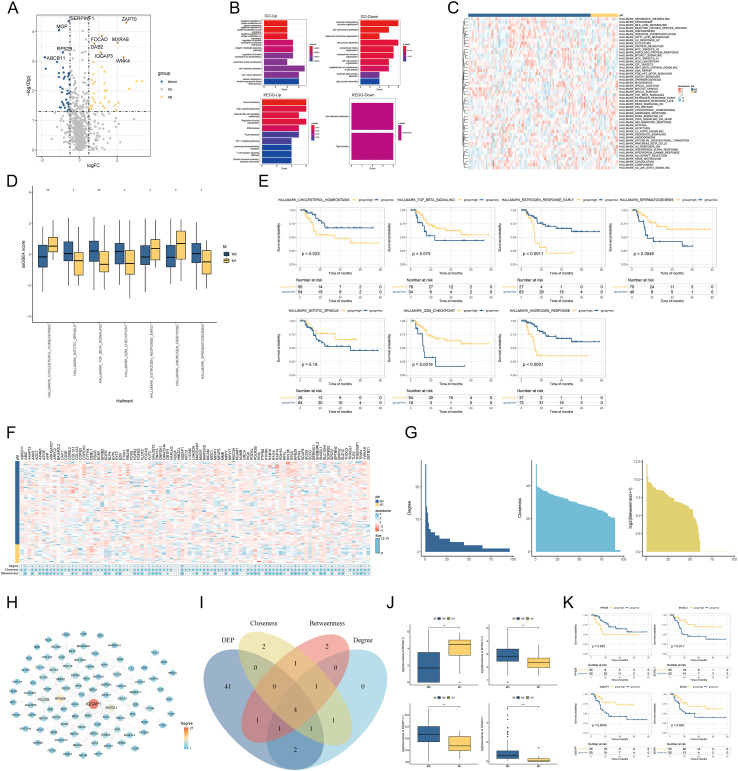
Differential extracellular matrix proteins and enrichment analysis, Identification of ECM hub proteins driving tumor metastasis. **(A)** Volcano plots of extracellular matrix proteins differentially expressed among pM subgroups; **(B)** GO and KEGG functional enrichment analysis of up-and down-regulated proteins; **(C)** Heatmap of the pathway enrichment results; **(D)** Intergroup differences in the pathway enrichment results; **(E)** Results of the prognostic analysis of the differential pathways between the groups; **(F)** Abundance, Degree, Closeness centrality, and Betweenness centrality of all proteins in the protein-protein interaction network; **(G)** Distribution of Degree, Closeness centrality, and Betweenness centrality of all proteins in the protein-protein interaction network; **(H)** Protein-protein interaction network; **(I)** Venn diagram of intersections; **(J)** Intergroup differential expression of hub proteins; **(K)** KM curve of hub proteins. (****p < 0.0001; ***p < 0.001; **p < 0.01; *p < 0.05).

Database analysis identified 1303 extracellular matrix proteins. Using histology data (PMC9522856), differential expression analysis across pM groups yielded 74 differentially expressed extracellular matrix proteins. Upregulated proteins (37 total) were defined as those significantly more abundant in the non-metastatic group(M0), while downregulated proteins were significantly less abundant in M0 (**Figure 1A**; **Supplementary Table 1**). GO and functional enrichment analyses of corresponding genes revealed distinct pathways for up- and downregulated proteins. Upregulated proteins were enriched in pathways such as negative regulation of protein localization to the plasma membrane, integrin-mediated signaling, Rap1 signaling, and natural killer cell-mediated cytotoxicity. Downregulated proteins were enriched in external encapsulating structural organization, cell-cell junction assembly, cell adhesion assembly, cell adhesion molecules, and tight junctions (**Figure 1B**). Hallmark enrichment scores, calculated from the protein abundance matrix (**Supplementary Figure 1A**), identified seven significantly enriched pathways differing across pM groups (**Figures 1C, D**). Prognostic analysis revealed that higher scores for HALLMARK_CHOLESTEROL_HOMEOSTASIS, HALLMARK_ESTROGEN_RESPONSE_EARLY, and HALLMARK_ANDROGEN_RESPONSE were associated with worse prognosis, while higher scores for HALLMARK_SPERMATOGENESIS and HALLMARK_G2M_CHECKPOINT correlated with better prognosis. HALLMARK_TGF_BETA_SIGNALING and HALLMARK_MITOTIC_SPINDLE showed no significant prognostic impact (**Figure 1E**). Differential expression analysis across pM groups identified 1535 proteins, and prognostic analysis across all proteins identified 734 prognostic-related proteins. The intersection of these sets yielded 282 prognostic-related differentially expressed proteins, from which the top 50 were selected for further analysis (**Supplementary Table 2**). Intersecting these with extracellular matrix-related proteins yielded 77 prognosis-related extracellular matrix proteins. A protein-protein interaction network, constructed using the top 50 prognostic-related differentially expressed proteins and the 77 prognosis-related extracellular matrix proteins, comprised 150 interactions (**Supplementary Table 3**). Analysis of degree, closeness, and betweenness centrality within this network identified four core hub proteins (**Figures 1F–I**). Further analysis demonstrated that RPS29 was significantly more abundant in M1 than M0, while IQGAP1, SH3GL1, and SCGN were more abundant in M0. Furthermore, high RPS29 abundance correlated with a worse prognosis, while low IQGAP1, SH3GL1, and SCGN abundance correlated with a poorer prognosis (**Figures 1J, K**).

### 3.2 Comprehensive single-cell transcriptomic analysis of stem cell subtypes and hub proteins

**Figure 2:**
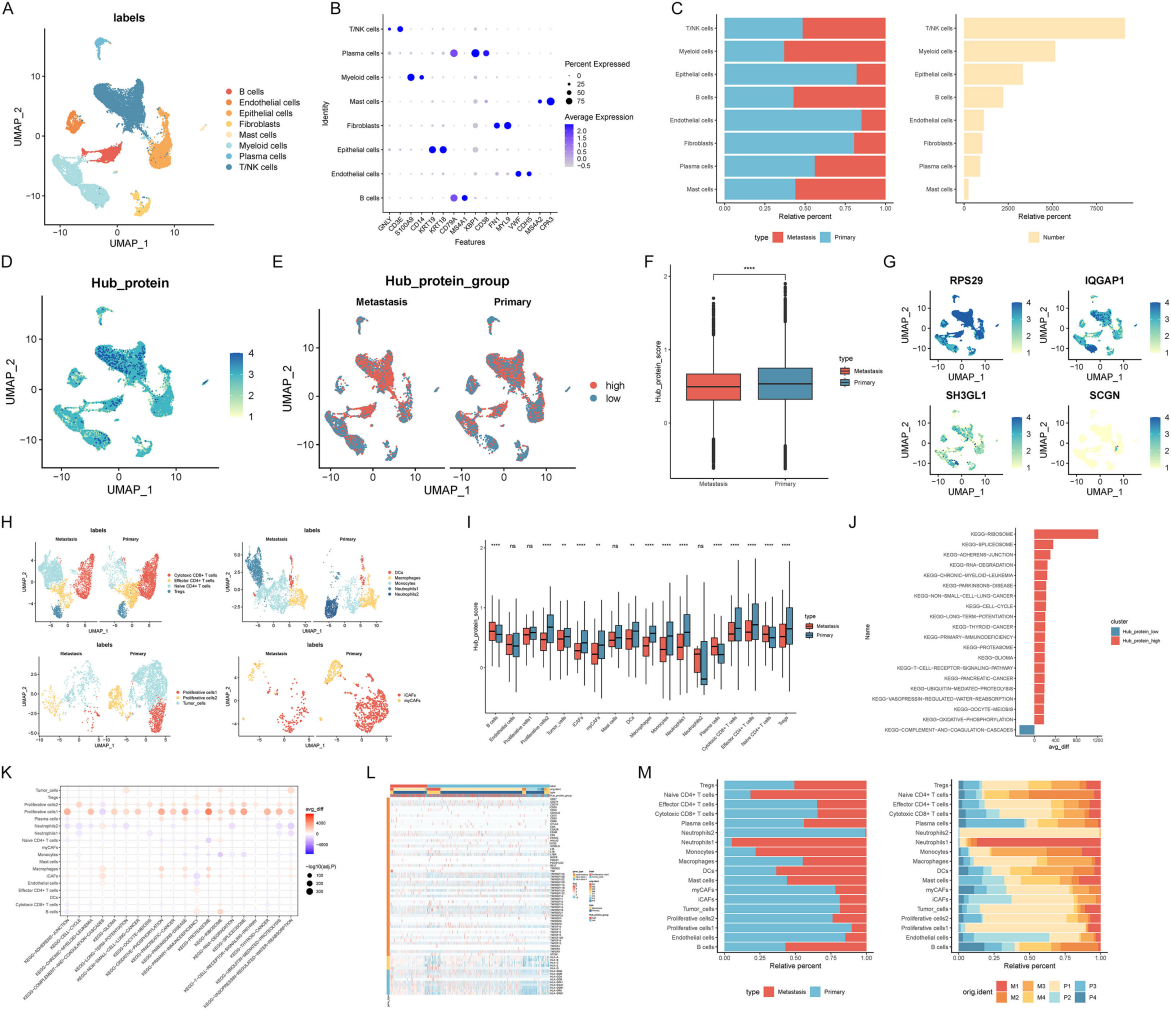
Single-cell transcriptome landscape and subtype analysis of gastric cancer, Functional enrichment analysis, expression heatmap of HLA genes, co-stimulatory molecules. **(A)** UMAP plot of cell types; **(B)** Expression bubble plots of markers for each cell type; **(C)** Percentage of cells in groups with different metastatic status and the number of cells; **(D)** UMAP plot of the enrichment score of key proteins; **(E)** UMAP plot of cells based on the enrichment scores of key proteins; **(F)** Comparison of key protein enrichment scores between metastasis status groups; **(G)** UMAP plots of key protein expression levels; **(H)** UMAP plots of cell subtypes; **(I)** Comparison of pivotal protein enrichment scores within each cell for metastasis status groups; **(J)** KEGG pathway enrichment scores based on high and low hub protein enrichment score groups; **(K)** KEGG pathway enrichment score statistics for cellular subtypes; **(L, M)**. Distribution of cellular subtypes across metastasis status groups and samples. **p < 0.01; ****p < 0.0001; ns, not significant.

Integrating two datasets, we analyzed four primary foci samples, two with metastases and two without. Single-cell sequencing identified eight initial cellular isoforms (**Supplementary Table 4**). Comparing cellular proportions between metastasis and non-metastasis samples revealed minimal differences in immune-like cell proportions, while epithelial cells, endothelial cells, and fibroblasts constituted larger proportions in non-metastasis samples (**Figures 2A–C**; **Supplementary Figures 1B–E**). Hub protein enrichment scores, calculated for each cell type and categorized as high or low, were significantly higher in T/NK cells and slightly lower in Myeloid cells, with a more even distribution across other cell types (**Figure 2D**). UMAP plots based on these score subgroups demonstrated higher T/NK and B cell scores in metastasis-free samples (**Figure 2E**), a finding confirmed by differential enrichment score analysis (**Figure 2F**). Given the observed heterogeneity in T/NK cells, myeloid cells, epithelial cells, and fibroblasts, we further identified subtypes within these populations (**Supplementary Figures 2A–E**; **Supplementary Table 5**). Examining the expression of four hub proteins (**Supplementary Figures 2F–I**), we found RPS29 highly expressed primarily in T/NK, B, and epithelial cells, and IQGAP1 highly expressed mainly in T/NK cells and some myeloid cells, with comparatively low expression of the remaining two genes across all cell types (**Figure 2G**).

T/NK cells were categorized into four subtypes, revealing a significant increase in naive CD4+ T cells within the metastatic group compared to the non-metastatic group, while the remaining subtypes were more prevalent in the non-metastatic group. Myeloid cells were classified into five subtypes, with Neutrophils1 and Monocytes primarily concentrated in the metastatic group, and Neutrophils2 mainly distributed in the non-metastatic group. Epithelial cells were identified as three subtypes, mainly distributed in the non-metastatic subgroup, particularly Proliferative cells1, which expressed both the tumor cell marker EPCAM and the proliferative cell marker MKI67. Fibroblasts, classified into two subtypes, were primarily located in the non-metastatic group (**Figure 2H**). Analysis of hub protein enrichment scores across cell types in metastatic and non-metastatic groups indicated higher scores in the non-metastatic groups for all cell types except B cells and Naive CD4+ T cells, which exhibited higher scores in the metastatic groups (**Figure 2I**).

KEGG enrichment scores were calculated for each cell, and the differences in scores between high and low hub protein enrichment score subgroups were analyzed. Among the top 20 enriched pathways, the high hub protein enrichment score subgroup was primarily enriched in KEGG-RIBOSOME, KEGG-SPLICEOSOME, and KEGG-ADHERENS-JUNCTION, while the low hub protein enrichment score group was mainly enriched in KEGG-COMPLEMENT-AND-COAGULATION-CASCADES (**Figure 2J**). At the cellular level, the top 20 pathways, displayed in a bubble plot, revealed that Proliferative cells1 were predominantly enriched in KEGG-PROTEASOME, KEGG-OXIDATIVE-PHOSPHORYLATION, and other pathways (**Figure 2K**; **Supplementary Figure 3A**). Expression of 60 T-cell co-stimulation-related genes (PMID: 37068318) and 15 HLA genes (PMID: 35533264) was visualized in heatmaps for Proliferative cells1 and Tumor_cells, showing lower HLA gene expression in some Tumor_cells (**Figure 2L**; **Supplementary Figure 3B**). Analysis of cell subtype distribution across metastatic subgroups and samples indicated that Neutrophils1 were mainly found in metastatic samples, particularly M1 samples, whereas Neutrophils2 were predominantly in non-metastatic samples, especially P1 samples (**Figure 2M**).

### 3.3 Characterizing tumor subtype differentiation and development based on hub protein expression within inferCNV subpopulations

**Figure 3:**
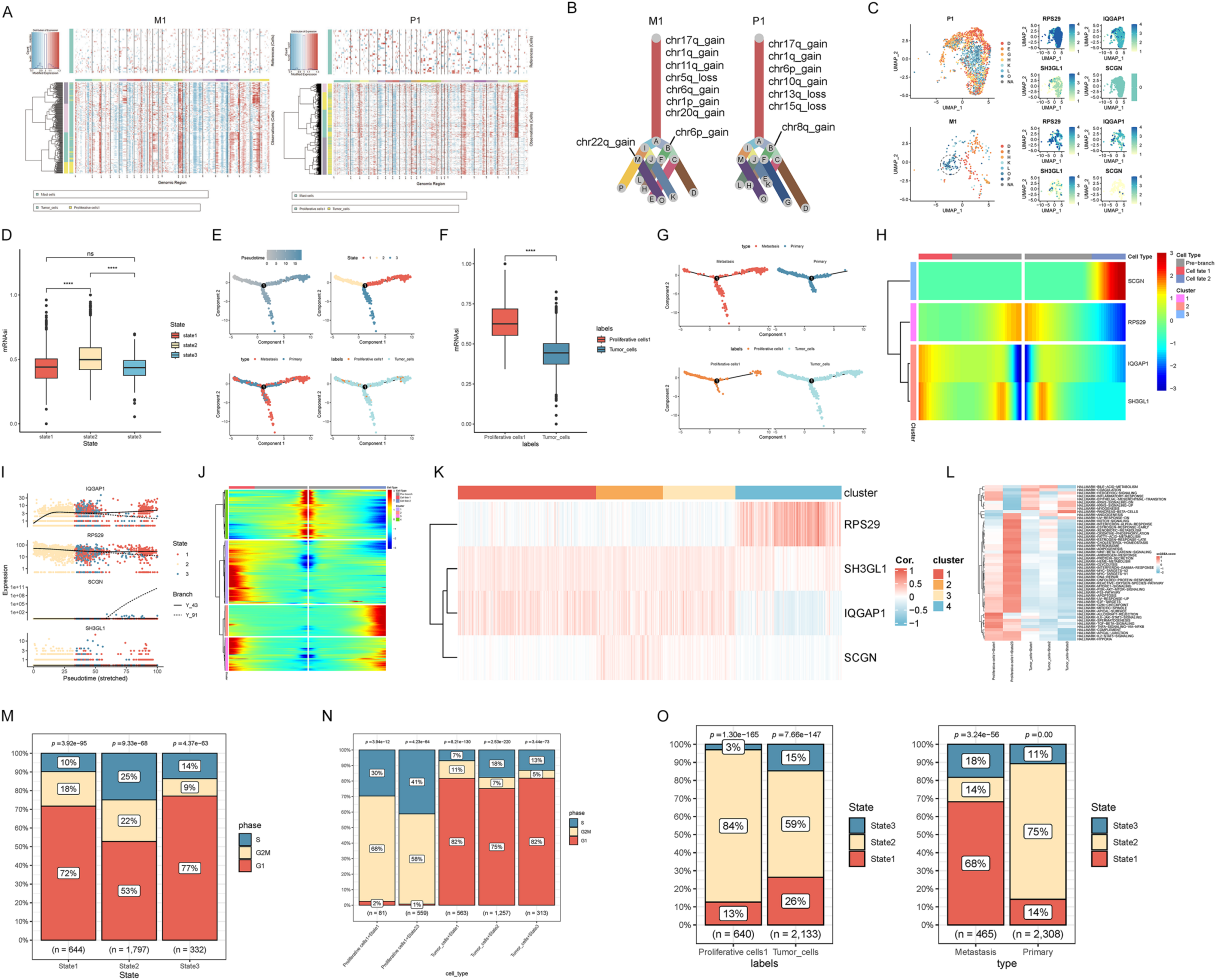
Characterizing tumor subtype differentiation and development based on hub protein expression within inferCNV subpopulations. **(A)** Heatmap of expression differences between M1 samples and P1 samples based on chromosomal regions; **(B)** Clonal tree representation of M1 samples and P1 samples based on CNV-predicted isoforms; **(C)** UMAP visualization of CNV-predicted isoforms and UMAP visualization of pivotal protein expression based on CNV predicted isoforms; **(D)** Stemness scores of different states; **(E)** Proposed model of tumor cell subtype evolution over time; **(F)** Stemness scores of tumor subtype cells; **(G)** Proposed temporal plots illustrating the relationship between metastasis grouping and cell subtype; **(H, I)**. Hub protein expression changes based on time are shown, in which Cell Type’s Pre-branch mainly refers to state2, Cell fate 1 refers to state1, Cell fate 2 mainly refers to state3, and Y_43 represents state1 and Y_91 represents state2 in Branch classification; **(J)** Expression of differentially expressed genes based on the proposed time series; **(K)** Correlation of gene expression between different subtypes and hub protein genes; **(L)** Functional enrichment analysis of tumor cell hallmarks integrating cell subtype and cell stage; **(M, N)** Cell cycle distribution within different cell types; **(O)**. Distribution of cell states across subtypes and metastasis groups. ****p < 0.0001; ns, not significant.

InferCNV analysis, initially performed on epidermal cells, endothelial cells, and fibroblasts, revealed substantial differential expression between Proliferative cells1 and Tumor_cells (**Supplementary Figure 3C**). Given the predominant origin of these cell types from M1 and P1 samples, respectively, CNV prediction was conducted separately for each sample. Expression heatmaps across chromosomal regions confirmed marked expression differences, suggesting a higher prevalence of CNV variants in these two cell types (**Figure 3A**). Subsequent CNV variant prediction and subtype identification facilitated the construction of clonal trees, each exhibiting seven branches representing distinct subtypes. Branches constituting less than 5% of the cell population in either sample were excluded from visualization. M1 samples exhibited predominant variants in chr11q_gain, chr5q_loss, chr6q_gain, chr1p_gain, and chr20q_gain, with branch B significantly enriched for chr6p_gain variants and branch M significantly enriched for chr22q_gain variants. CNV variant frequencies in P1 samples were significantly lower than those in M1 samples, indicating potential sample shifts with increased CNV variant frequencies (**Supplementary Table 6**). P1 samples primarily displayed variants in chr6p_gain, chr10q_gain, chr13q_loss, and chr15q_loss, with branch B significantly enriched for chr8q_gain variants (**Figure 3B**). Chi-square tests comparing branch frequencies between P1 and M1 samples revealed significantly higher frequencies of branches E and P in M1 samples, while branch G was significantly more frequent in P1 samples (**Supplementary Table 7**). UMAP plots illustrating cellular branch distributions demonstrated a significant difference in the distribution of branch P in M1 samples compared to RPS. Furthermore, the distribution of branch P in M1 samples exhibited greater overlap with high expression areas of RPS29 and IQGAP1, suggesting a potential association between these genes and metastatic grouping (**Figure 3C**).

To investigate the regulatory patterns and mechanisms by which tumor hub proteins drive metastasis, we focused on Proliferative cells1 and Tumor_cells, as prior analysis indicated their heightened malignancy and potential role in metastasis. Time-series analysis of these cell types’ expression matrices identified three distinct states. STATE2 exhibited the highest degree of stemness, suggesting its position at the beginning of the developmental trajectory, thus serving as our starting point (**Figure 3D**). Trajectory analysis of cells, grouped by metastatic potential, revealed that non-metastatic cells were primarily located in STATE2 and the other two states, while metastatic cells were predominantly found at the end of the latter two states, with a small presence in STATE2. This suggested that metastatic trajectories originated in STATE2 and progressed towards STATE1 and STATE3, mirroring our temporal analysis. Proliferative cells1 were mainly concentrated in STATE2, with a small fraction in late STATE1, while tumor cells were distributed across all states. The significantly higher stemness of Proliferative cells1 compared to Tumor_cells suggests that Proliferative cells1 may be associated with metastasis initiation (**Figure 3E-G**). Analysis of the four hub proteins’ expression across the trajectories, focusing on RPS29 and IQGAP1 due to low expression of SCGN and SH3GL1, showed that IQGAP1 expression gradually increased and stabilized with progression from low levels in early STATE2, with a slight increase at the end of STATE1 and a significant decrease at the end of STATE3. RPS29 was highly expressed throughout, with a slight decrease in the late stages and a significant decrease at the end of STATE3 (**Figures 3H–I**; **Supplementary Figure 3D**). Differential gene expression analysis across time, using the top 500 genes for subtype identification and heatmap visualization, yielded four gene expression subtypes. KEGG enrichment analysis revealed that cluster 1 genes, highly expressed in STATE1, were enriched in pathways like antigen processing and presentation and allograft rejection. Cluster 2 genes, highly expressed in STATE3, were enriched in protein processing in the endoplasmic reticulum and MAPK signaling pathways. Cluster 3 genes, also highly expressed in STATE3, were enriched in MAPK signaling pathways. Cluster 4 genes, highly expressed in STATE1 and STATE3, were enriched in ferroptosis and autophagy pathways. (**Figure 3J**; **Supplementary Figure 3E**; **Supplementary Table 8**).

Correlation analysis between isoform genes and hub proteins revealed a strong association of RPS29 with the cluster 4 gene group, while IQGAP1 exhibited a high correlation with all other gene groups (**Figure 3K**). Integrating cell subtype and temporal groupings, the low cell count in Proliferative cells1+State3 necessitated its merger with Proliferative cells1+State2, creating a combined Proliferative cells1+State23 group. Subsequent Hallmark functional enrichment analysis demonstrated elevated EPITHELIAL-MESENCHYMAL-TRANSITION pathway scores in state 2 and 3 cells, particularly Proliferative cells1+State1 and Tumor_cells+State1, suggesting an association between state 1 and metastasis initiation. Additionally, Proliferative cells1+State2 showed enrichment in NOTCH-SIGNALING, OXIDATIVE-PHOSPHORYLATION, and WNT-BETA-CATENIN-SIGNALING pathways (**Figure 3L**). Cell cycle analysis indicated a predominance of G1 phase cells across all three states, though state 2 exhibited a higher proportion of G2M/S phase cells, indicative of increased cell division activity. This observation was reinforced by cell subtype analysis, which revealed a majority of Proliferative cells1 in the G2M/S phase, consistent with rapidly dividing malignant tumor cells (**Figures 3M, N**). Finally, analysis of state distribution across cell subtypes and metastasis groupings showed a concentration of Proliferative cells1 in state 2, while metastatic original samples were predominantly represented in state 1, and non-metastatic samples in state 2, further supporting the association of state 1 cells with metastasis (**Figure 3O**).

### 3.4 Bulk prognostic analysis

**Figure 4:**
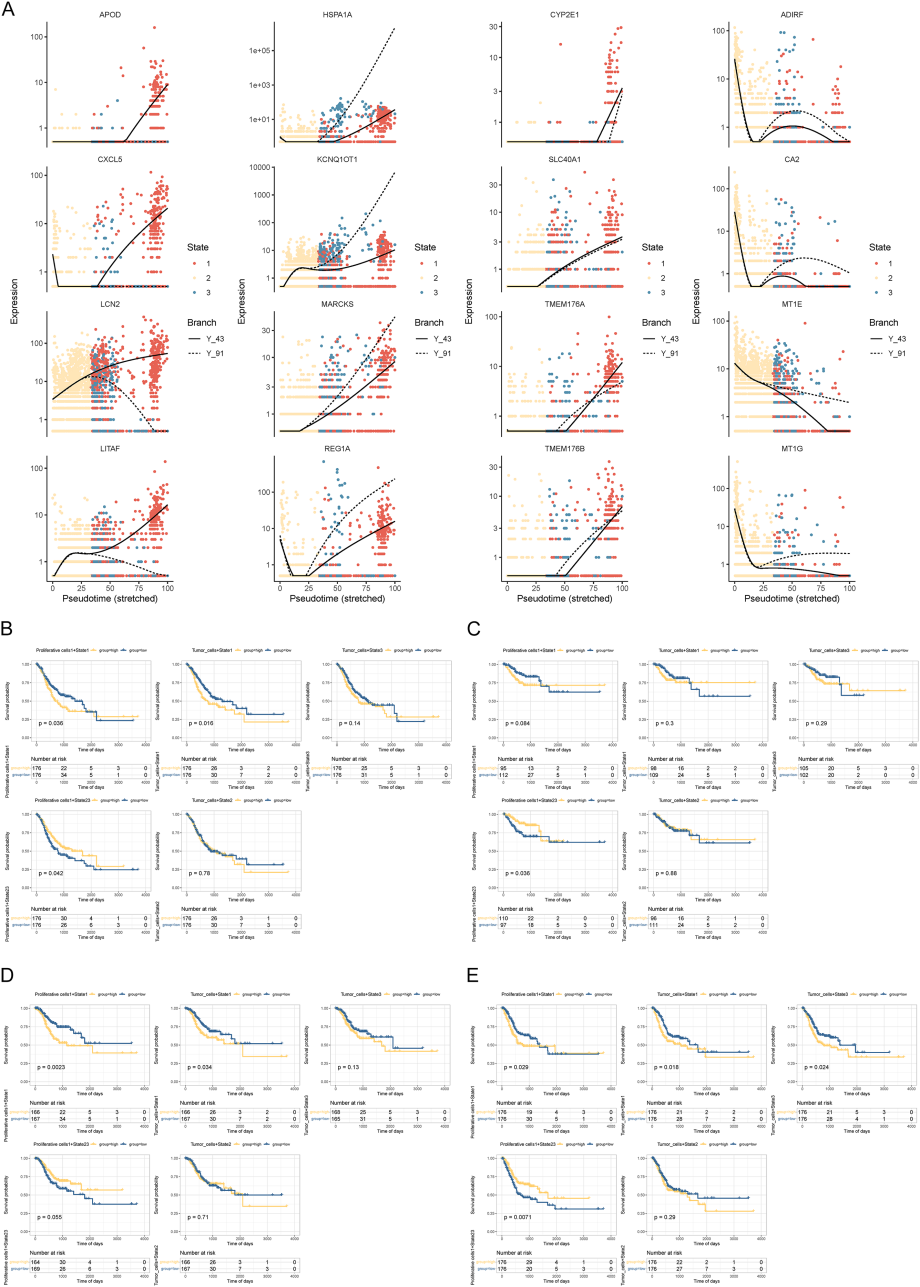
Prognostic analysis. **(A)** Expression of genes with typical expression trends in the four isoforms of time-associated differential genes; **(B–E)** Prognostic analysis based on OS, DFI, DSS, and PFI.

To illustrate the distinct expression patterns across the four molecular subtypes, scatter plots were generated using the top 500 differentially expressed genes identified from the top 500 list related to timing (**Figure 4A**). Subsequently, utilizing signature genes characteristic of Proliferative cells1 and Tumor_cells at various time points, enrichment scores were calculated within the Cancer Genome Atlas Stomach Adenocarcinoma (TCGA-STAD) cohort. Subsequent survival analyses revealed significant associations between Proliferative cells1 in State1 and poorer OS, DSS, and PFI; conversely, Proliferative cells1 in State2/3 correlated with worse OS, DFI, and PFI, but in this instance, lower enrichment scores were associated with worse outcomes. Finally, tumor cells in State1 demonstrated a significant association with worse OS, DSS, and PFI with higher enrichment scores indicative of poorer prognosis (**Figures 4B–E**).

### 3.5 Differential activation of tumor metastasis-related transcription factors

**Figure 5:**
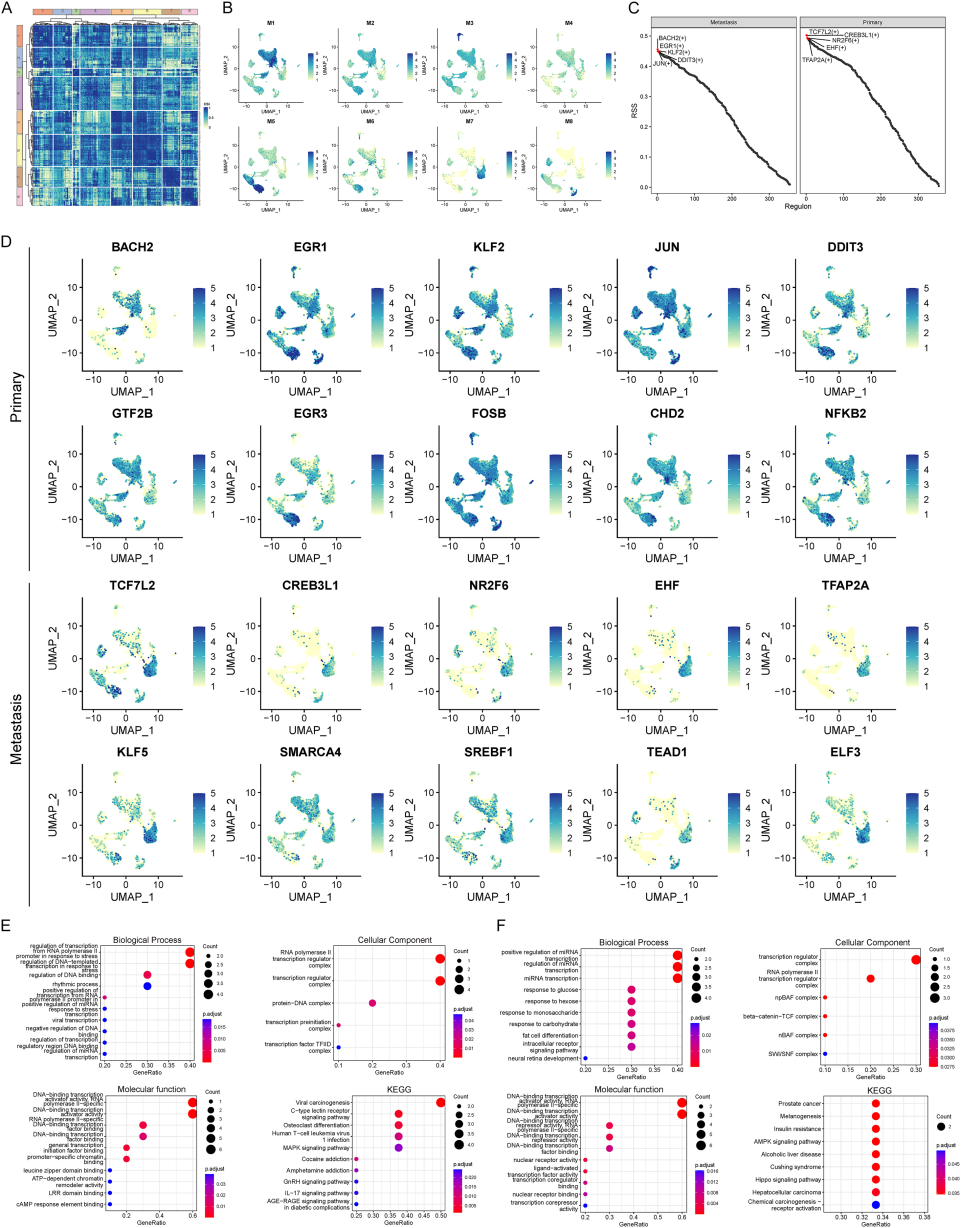
Differential activation of transcription factors in metastatic vs. non-metastatic groups. **(A)** Heatmap of transcription factor CSI values and identification of modules; **(B)** UMAP visualization based on mean CSI values of modules; **(C)** RSS sorting based on high and low NMAS groups; **(D)** UMAP visualization of transcription factor activity in metastasis and non-metastasis subgroups. **(E, F)** Functional enrichment analysis of transcription factors in **(E)** non-metastasis and **(F)** metastasis subgroups.

Transcription factor activities were further calculated for all cells, and CSI values and RSS values were calculated from AUC values. Eight regulatory modules were identified based on CSI values (**Figure 5A**), and a UMAP plot, based on the mean CSI scores within each module, revealed significant differences in differentiation degree between modules, with distinct mean CSI values observed across the UMAP plot (**Figure 5B**). Intergroup-specific transcription factors were identified for metastatic and non-metastatic samples, and RSS ranking maps were generated. BACH2, EGR1, KLF2, DDIT3, and JUN were the top five RSS-ranked transcription factors in the metastatic group, while TCF7L2, CREB3L1, NR2F6, EHF, and TFAP2A were the top five in the non-metastatic group (**Figure 5C**). The top ten RSS-ranked transcription factors for each group were designated as group-specific, and their AUC activities were visualized via UMAP. Non-metastatic group-specific transcription factors exhibited higher activity in most B cells, T/NK cells, some fibroblasts, and myeloid cells, whereas metastatic group-specific transcription factors showed higher activity in most epithelial cells (**Figure 5D**). Functional enrichment analyses were performed on these group-specific transcription factors. In the non-metastatic group, enriched Gene Ontology Biological Processes (BP) included regulation of transcription from the RNA polymerase II promoter and DNA-templated transcription in response to stress; enriched Cellular Components (CC) included RNA polymerase II transcription regulator complex; and enriched Molecular Functions (MF) included DNA-binding transcription activator activity. Enriched KEGG pathways included viral carcinogenesis and the C-type lectin receptor signaling pathway. In the metastatic group, enriched BP included positive regulation of miRNA transcription; enriched CC included transcription regulator complex; and enriched MF included DNA-binding transcription activator activity. Enriched KEGG pathways included melanogenesis and the AMPK signaling pathway (**Figures 5E, F**).

### 3.6 Single-cell analysis reveals differential intercellular communication and potential therapeutic targets in metastasis

**Figure 6:**
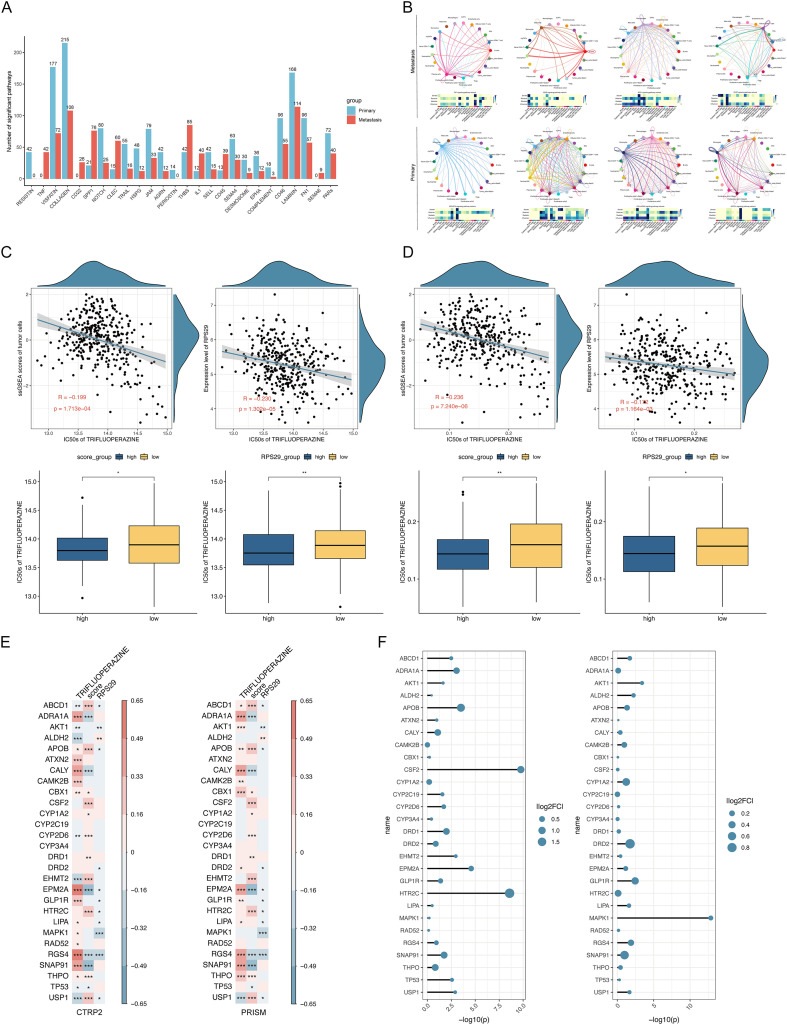
Analysis of Cellular Communication, Pathway Enrichment, and Drug Response Prediction. **(A)** Number of significant differences in intercellular communication between metastatic subgroups. **(B)** The top four significantly enriched pathways exhibited differential activity. **(C)** Drug response prediction results based on the CTRP2 database. **(D)** Drug response prediction results based on the PRISM database. **(E, F)** Correlation analyses between drug-interacting gene expression and drug IC50 values, pathway enrichment scores, and RPS29 expression. Differential expression of drug-interacting genes between pathway enrichment score subgroups and RPS29 expression subgroups is also shown.

Cell communication analysis, performed across all cells, revealed specific interactions detailed in **Supplementary Table 9**. These interactions were subsequently aggregated at the pathway level. In metastatic subgroups, intercellular interactions mapped 89 signaling pathways, while 92 pathways were identified in non-metastatic subgroups. Differences in pathway enrichment between these groups were assessed (**Figure 6A**; **Supplementary Table 10**), and the four pathways exhibiting the greatest intergroup variation were selected for network visualization and importance heatmap display. Metastatic subgroups showed significant enrichment in TNF, CD22, SPP1, and CLEC pathways. Notably, the TNF pathway, primarily involving Proliferative cells1+State1, Monocytes, Tregs, and Neutrophils1, was predominantly mediated by Proliferative cells2. Conversely, non-metastatic subgroups were significantly enriched in RESISTIN, VISFATIN, COLLAGEN, and NOTCH pathways. RESISTIN interactions primarily occurred between Monocytes and Macrophages, while the NOTCH pathway mainly involved epidermal cells, endothelial cells, and fibroblasts (**Figure 6B**). These findings highlight distinct cellular communication preferences between metastatic and non-metastatic subgroups at the pathway level.

Based on prior analysis, we identified Proliferative cells1+State1 and Tumor_cells+State1 as metastasis-associated cell types. We then combined the gene sets characterizing these two groups and calculated an enrichment score, stratifying samples into high- and low-enrichment groups based on the median score. Similarly, given the inference that RPS29 is a metastasis-associated hub protein, we also divided samples into high- and low-expression groups according to the median RPS29 expression. Predicting TCGA-STAD sample drug response IC_50_ values using CTRP2 and PRISM database information and integrating the results revealed a significant negative correlation, specifically between TRIFLUOPERAZINE IC_50_ values and both RPS29 expression and the aforementioned enrichment score, suggesting greater TRIFLUOPERAZINE sensitivity in patients within the high-expression/high-enrichment subgroups (**Figures 6C, D**). Subsequent database analysis identified 28 genes known to interact with TRIFLUOPERAZINE. Further correlation analysis of these interacting genes’ expression with TRIFLUOPERAZINE IC_50_, enrichment score, and RPS29 expression revealed significant correlations for ABCD1, APOB, EPM2A, RGS4, and USP1 across all three metrics (**Figures 6E, F**).

### 3.7 RPS29 is highly expressed in gastric cancer and promotes gastric cancer migration, invasion, and EMT

**Figure 7:**
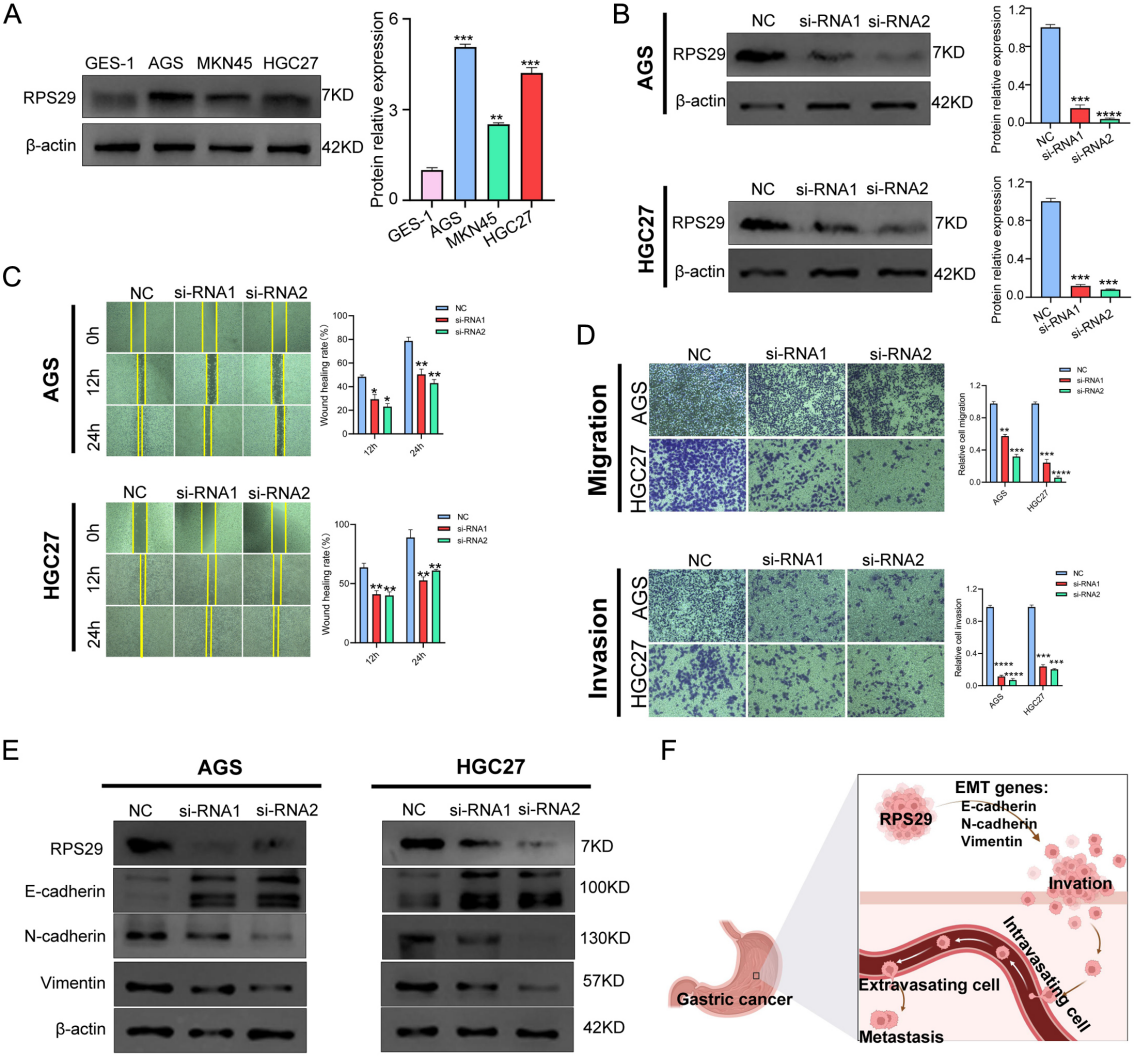
RPS29 is associated with gastric cancer cell metastasis. **(A)** RPS29 protein expression was higher in three gastric cancer cell lines (AGS, MKN45, HGC27) than in normal human gastric mucosal epithelial cells HES-1; **(B)** Knockdown groups were constructed in the highly expressed gastric cancer cell lines AGS, HGC27; **(C)** Scratch assay showed that wound healing was slowed down in AGS, HGC27 cell lines after knockdown of RPS29; **(D)** Transwell assay revealed that after knockdown of PS29, AGS, HGC27 cell lines had diminished motility invasion ability; **(E, F)** RPS29 knockdown resulted in increased expression of E-calmodulin and decreased expression of waveform protein and N-calmodulin, and RPS29 regulated these proteins to drive the EMT process and promote metastasis of gastric cancer cells. **p < 0.01; ***p < 0.001; ****p < 0.0001.

Database analysis revealed that RPS29 may play a key role in the metastatic process of gastric cancer, while current reported studies are lacking. The higher expression of RPS29 protein in gastric cancer cell lines compared with normal human gastric mucosal epithelial cells was first identified by *in vitro* cellular experiments (**Figure 7A**). To explore the role of RPS29 in the malignancy of gastric cancer, transient transfection knockdown was performed in AGS and HGC27, gastric cancer cell lines with high expression of RPS29 (**Figure 7B**). Subsequent wound healing assays showed that knockdown of RPS29 significantly inhibited cell migration in AGS and HGC27 (**Figure 7C**). In further transwell migration and invasion assays, it was found that the number of migrating and invading cells in AGS and HGC27 was lower in the RPS29 knockdown group than in the control group (**Figure 7D**). Epithelial-mesenchymal transition (EMT) is a biological process that enables polarized epithelial cells to acquire mesenchymal characteristics, thereby promoting tumor migration and invasion. Therefore, it was investigated whether RPS29 expression affects EMT. Protein blotting results showed that the knockdown of RPS29 significantly decreased the expression of mesenchymal markers waveform protein and N-calmodulin but increased the expression of epithelial marker E-calmodulin in AGS and HGC27 (**Figure 7E**). The above experimental results indicated that RPS29 might promote migration, invasion, and EMT of gastric cancer cells (**Figure 7F**).

### 3.8 Technology roadmap

**Figure 8:**
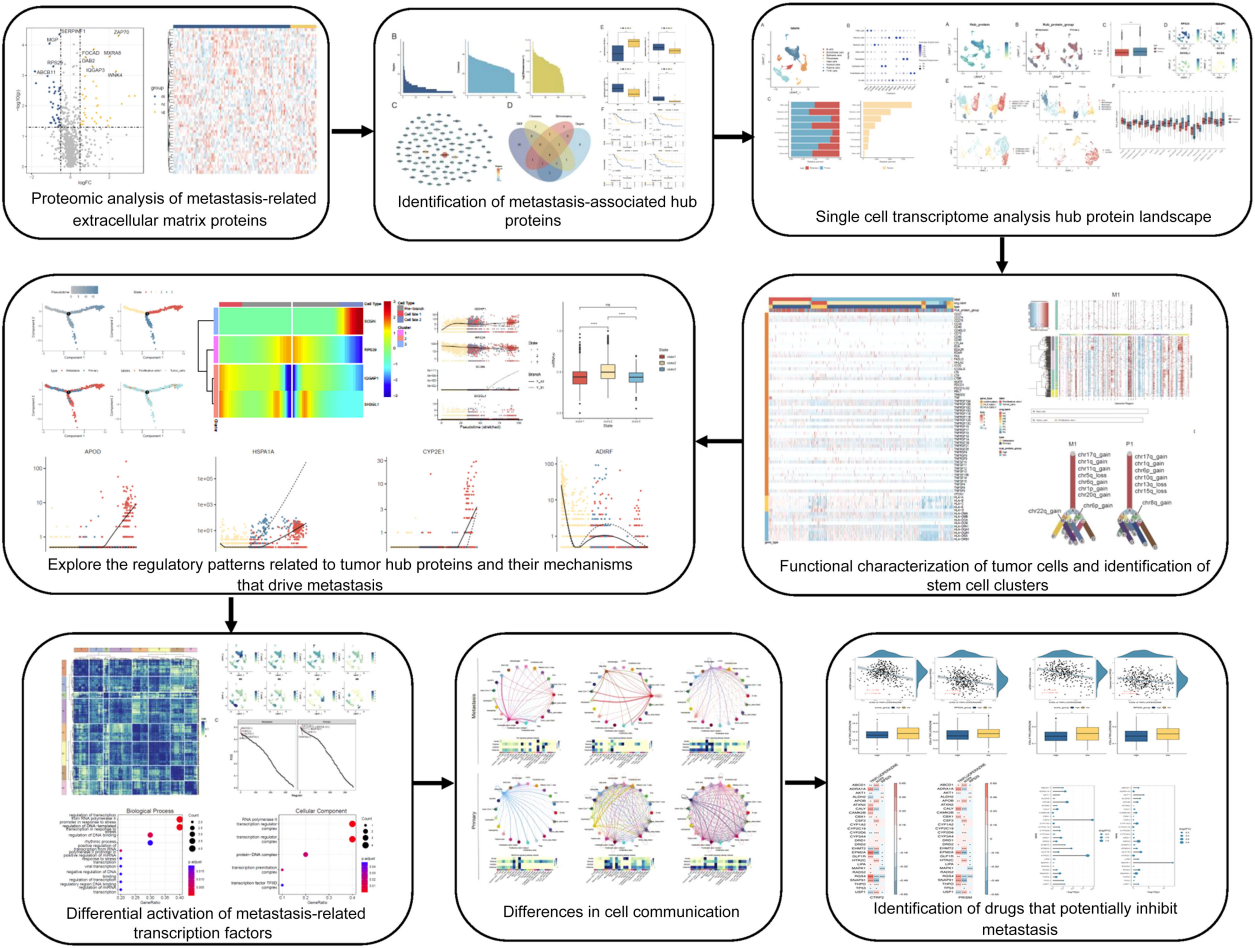
Technology roadmap.

The technology roadmap (**Figure 8**) clearly illustrates the complete logical chain and key decision points. Data acquisition (proteomics, single-cell transcriptomics), Differential analysis and prognostic screening,PPI network construction and hub protein identification, Single-cell expression and functional association analysis, *In vitro* functional validation focusing on RPS29 (migration, invasion, EMT).

## 4 Discussion

Gastric cancer, a highly lethal malignancy, exhibits a concerning rise in incidence. The advanced-stage disease is frequently characterized by significant metastasis (37), a primary driver of poor patient outcomes. Current treatments for gastric cancer metastasis lack clearly defined targets and effective mechanisms, limiting improvements in patient survival and quality of life. Therefore, a comprehensive understanding of the signaling pathways and key molecules involved is crucial for developing targeted therapies and improving clinical outcomes.

A particularly intriguing finding from our cell-cell communication analysis was the significant enrichment of the NOTCH signaling pathway in the tumor microenvironment of the non-metastatic group. This result may seem counterintuitive given that NOTCH signaling has been reported to have oncogenic roles in some gastric cancer contexts (38). However, the function of the NOTCH pathway is exquisitely context-dependent, contingent on the specific ligands, receptors, downstream targets, and crosstalk with other pathways present in a given cellular environment (39, 40). Our data strongly support a tumor-suppressive or metastasis-suppressive role for NOTCH in the specific context of our patient cohort.We hypothesize that in these primary tumors, active NOTCH signaling may function to maintain a more differentiated epithelial state and enforce tissue homeostasis, thereby acting as a barrier to the dedifferentiation and cellular plasticity required for metastasis. For instance, canonical NOTCH signaling is known to promote cell fate decisions that lead to differentiation and can induce cell cycle arrest, both of which are anti-metastatic functions. Its enrichment in the non-metastatic group suggests that the loss or downregulation of this homeostatic NOTCH network may be a key event that permits cells to embark on a metastatic trajectory. This is further supported by the concomitant enrichment of pro-inflammatory and pro-invasive pathways such as TNF, SPP1, and CLEC in the metastasis-positive group, suggesting a functional switch away from homeostatic signaling towards pathways that actively promote invasion and dissemination (41, 42). Future functional studies are warranted to experimentally validate this metastasis-suppressive role of NOTCH signaling in GC.

Using drug response data from the CTRP2 and PRISM databases to predict IC_50_ values in TCGA-STAD samples, we found that TRIFLUOPERAZINE’s IC_50_ was significantly correlated with RPS29 expression and enrichment scores. Specifically, a significant negative correlation suggests that patients with high expression of RPS29 may be more sensitive to TRIFLUOPERAZINE. Studies have shown that trifluoperazine, an FDA-approved antipsychotic, reduces cerebral ischemia/reperfusion injury in rats via the AMPK signaling pathway and mitochondria-dependent mechanisms (43). Trifluoperazine, a calmodulin inhibitor, has demonstrated anti-cancer potential across various cancer types. In osteosarcoma cells, it activates the AMPK/mTOR/ULK1 signaling pathway to induce mitochondrial autophagy (44). In oral cancer, trifluoperazine induces SLC7A11/GPX4-mediated ferroptosis via the ROS/autophagy pathway (45). It also prevents phenotypic transformation and improves survival in a mouse model of glioblastoma (46). In lung cancer cells, trifluoperazine inhibits growth and overcomes erlotinib resistance in EGFR-mutant cells by inhibiting the Wnt/β-catenin signaling pathway and decreasing the expression of the cancer stem cell marker CD44/CD133 (47). In breast cancer, it inhibits the growth of triple-negative breast cancer by inducing cell cycle arrest and apoptosis in the G0/G1 phase (48). In colorectal cancer, trifluoperazine inhibits tumor growth by inducing apoptosis and cell cycle arrest (49). In pancreatic ductal adenocarcinoma (PDAC), trifluoperazine inhibits tumor growth by downregulating NUPR1 gene expression (50). As a calmodulin inhibitor, trifluoperazine regulates cell proliferation, migration, and apoptosis through multiple signaling pathways, including reducing Bcl-2 protein levels and increasing the expression of caspase 8 and Bax, thereby promoting apoptosis (51).

A primary contribution of this work is the powerful new insight gained through the synergistic integration of publicly available, yet previously disconnected, proteomic and single-cell transcriptomic datasets. By developing a computational pipeline to link key differentially expressed ECM proteins from bulk tissue analysis to the cellular resolution of scRNA-seq, we were able to generate novel, testable hypotheses about the specific cell states and trajectories associated with a pro-metastatic microenvironment. This hypothesis-generating strength, derived from re-analyzing existing data through a new analytical lens, represents the core novelty of our approach.

We note that while previous single-cell studies in gastric cancer catalog cellular heterogeneity, our work is distinct in its prior filtration of targets using metastasis-associated ECM proteins from proteomics. This allows us to specifically interrogate how these predefined key proteins map onto malignant cell trajectories. Our cell subtype analysis further identified Proliferative cells1 and Tumor_cells as key entities, and trajectory analysis suggested their progression from a high-stemness state (STATE2).

Four hub proteins—RPS29, IQGAP1, SH3GL1, and SCGN were identified through screening. RPS29 was prioritized for *in vitro* validation due to its significantly higher expression in the metastatic group (M1) compared to the non-metastatic group (M0), with this elevated expression correlating with poorer prognosis in terms of both strength and direction of association. Conversely, IQGAP1, SH3GL1, and SCGN exhibited higher expression in the M0 group, with their low expression correlated to poor prognosis. Regarding network centrality, within the constructed protein-protein interaction network, RPS29 demonstrated high connectivity with all other hub proteins. Its core network position suggests a potentially pivotal role in regulatory networks. Potential association with metastasis-related cell subtypes and EMT. Single-cell trajectory analysis revealed sustained high expression of RPS29 in the STATE2 state, characterized by high pluripotency and potential relevance to metastasis initiation. Although experimental validation focused on RPS29, the remaining proteins IQGAP1, SH3GL1, and SCGN may participate in shaping the metastatic microenvironment through distinct pathways, warranting future investigation.

Although our bioinformatics study yielded insightful preliminary findings, it is important to acknowledge its limitations. A major limitation of our study is the exclusive focus on primary tumor tissues. While we successfully identified ECM signatures within primary tumors that are associated with metastatic potential, we did not analyze the ECM composition of metastatic lesions themselves. It is well established that the microenvironment of a metastatic site can differ significantly from that of the primary tumor, and that cancer cells must adapt to this new niche. Therefore, our findings reflect the ECM state that facilitates the initial steps of metastasis (local invasion and intravasation) but do not provide insight into the processes of extravasation and colonization at a distant site. A direct comparative analysis of paired primary and metastatic gastric cancer tissues is a crucial next step to fully delineate the dynamic evolution of the ECM throughout the entire metastatic cascade.

## 5 Conclusions

In conclusion, this study establishes a multi-omics integration and experimental validation framework that can be extended to other solid tumours, systematically elucidating the ECM-driven mechanisms of metastasis. Future research should leverage machine learning and deep learning models to further dissect the complexities of the tumor microenvironment; these advanced methods hold the promise of identifying more robust and subtle biomarkers. Regarding RPS29, subsequent research will establish an animal model of gastric cancer metastasis for *in vivo* experiments and investigate whether targeting RPS29 can enhance the efficacy of existing chemotherapy or targeted therapies, thereby providing evidence for clinical translation.

## Data Availability

The datasets presented in this study can be found in online repositories. The names of the repository/repositories and accession number(s) can be found in the article/**Supplementary Material**.

## Supplementary material

Supplementary Figure 1 **(A)** Differential Hallmark pathways between pM groups for all samples based on proteomic data; **(B, C)**. Single-cell data preprocessing results; **(D)**. UMAP plot presentation of single-cell data based on sample grouping and raw cluster grouping; **(E)**. UMAP visualization of marker expression for different cell types.

Supplementary Figure 2 **(A–E)** UMAP plots and bubble plots showing the expression of individual subtype markers after subtype identification of T/NK cells, Myeloid cells, Epithelial cells, and Fibroblasts; **(F)**. Average expression levels of all intracellular hub proteins from single-cell data are shown; **(G–J)**. UMAP plot demonstrating the expression of hub proteins in T/NK cells, Myeloid cells, Epithelial cells, and Fibroblasts.

Supplementary Figure 3 **(A)** Differential KEGG enrichment analysis across cell types; **(B)** Expression of HLA genes and T-cell co-stimulation-related genes in all cells; **(C)** Differential gene expression analysis based on chromosomal location in epidermal cells, endothelial cells, and fibroblasts; **(D)** Expression changes of hub protein-coding genes across the proposed time series and differential expression between transfer subgroups; **(E)** KEGG pathway enrichment analysis of genes exhibiting differential expression across the time series of different isoforms.
