# Supplementary figures and images for: ECM remodeling-associated immune signatures and hub proteins: predictive markers and therapeutic targets for metastatic gastric cancer
Source: Front Immunol. 2026 Feb 6;17:1765095. doi: 10.3389/fimmu.2026.1765095 (PMC12920572; doi:10.3389/fimmu.2026.1765095)

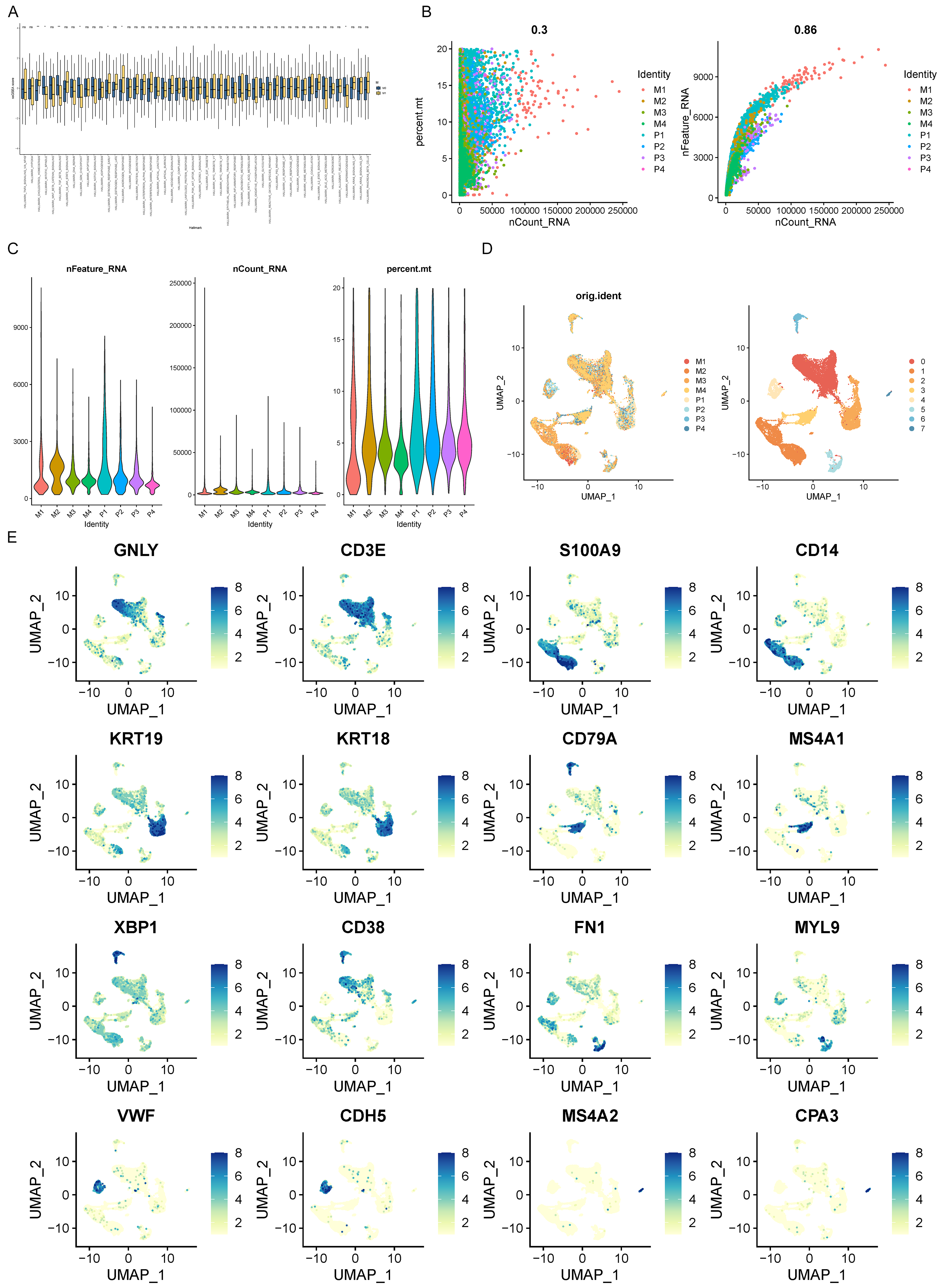

Supplement: Supplementary Figure 1 — (A) Differential Hallmark pathways between pM groups for all samples based on proteomic data; (B, C). Single-cell data preprocessing results; (D). UMAP plot presentation of single-cell data based on sample grouping and raw cluster grouping; (E). UMAP visualization of marker expression for different cell types. [file Image1.tif]

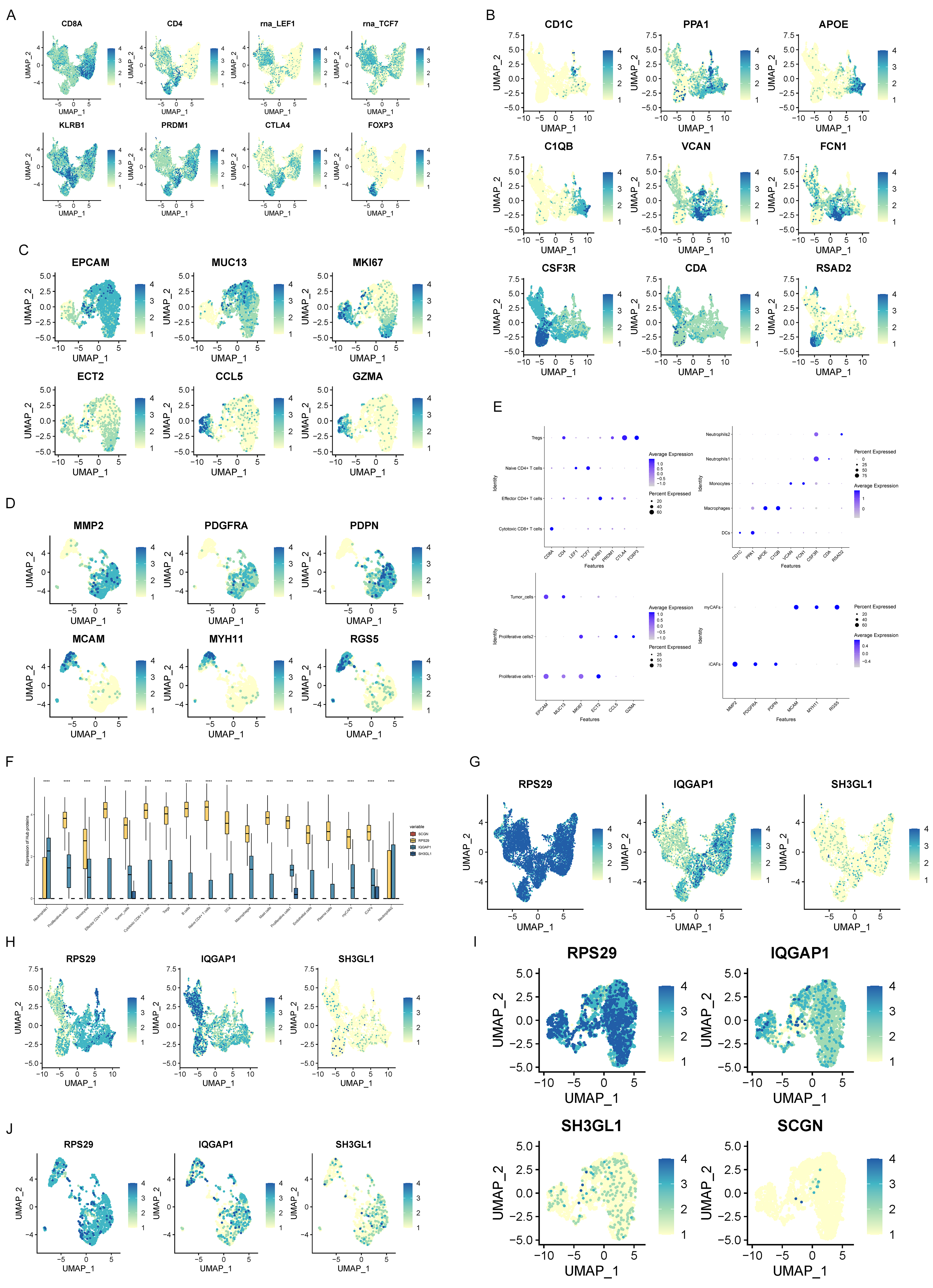

Supplement: Supplementary Figure 2 — (A–E) UMAP plots and bubble plots showing the expression of individual subtype markers after subtype identification of T/NK cells, Myeloid cells, Epithelial cells, and Fibroblasts; (F). Average expression levels of all intracellular hub proteins from single-cell data are shown; (G–J). UMAP plot demonstrating the expression of hub proteins in T/NK cells, Myeloid cells, Epithelial cells, and Fibroblasts. [file Image2.tif]

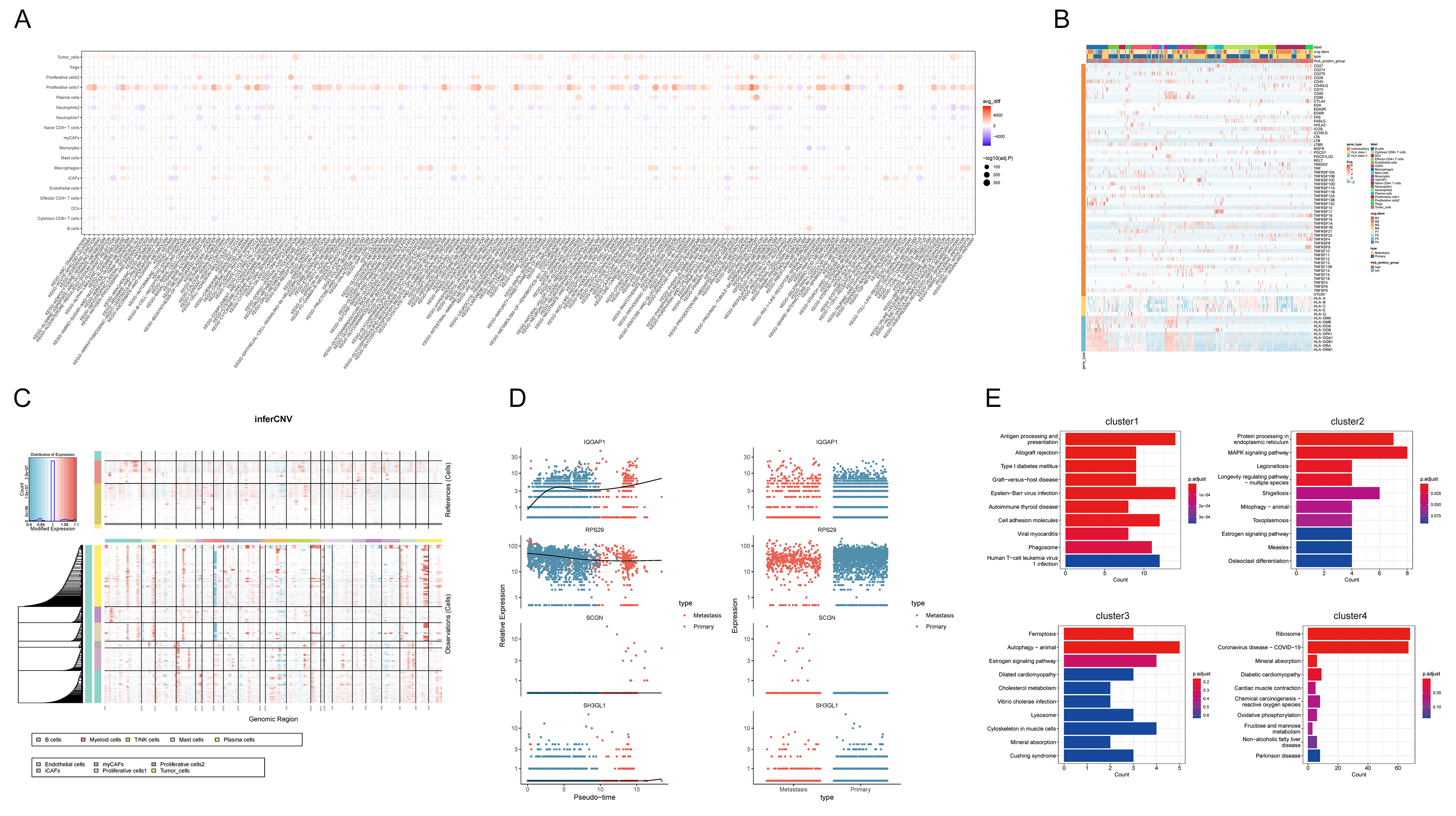

Supplement: Supplementary Figure 3 — (A) Differential KEGG enrichment analysis across cell types; (B) Expression of HLA genes and T-cell co-stimulation-related genes in all cells; (C) Differential gene expression analysis based on chromosomal location in epidermal cells, endothelial cells, and fibroblasts; (D) Expression changes of hub protein-coding genes across the proposed time series and differential expression between transfer subgroups; (E) KEGG pathway enrichment analysis of genes exhibiting differential expression across the time series of different isoforms. [file Image3.tif]
